# P36 A case of refractory IBD-related scleritis

**DOI:** 10.1093/rap/rkab068.035

**Published:** 2021-10-19

**Authors:** Lucy Paterson-Brown, Alexandra Jones, Nick Wilkinson

**Affiliations:** Evelina London Children's Hospital, London, United Kingdom

## Abstract

**Case report - Introduction:**

Scleritis is severe vision-threatening scleral inflammation, commonly presenting with a red, painful eye and can be classified using the Watson system. Posterior scleritis may present with associated anterior uveitis in many cases. Steroid therapy is often successful initially; however, further immunomodulation is usually required to control subsequent episodes which can be challenging. We present an 18-year-old, Caucasian girl with a left eye, sight-threatening, steroid-dependent posterior uveitis who required escalation of treatment to tocilizumab before inflammation could be suppressed.

**Case report - Case description:**

Our patient was diagnosed elsewhere with ulcerative colitis at the age of 12 and subsequently developed recurrent episodes of uveitis and scleritis which could be controlled with topical steroids. At the age of 16 she presented with an inflammatory arthritis and was treated with intravenous methylprednisolone before commencing sulfasalazine therapy. Due to persistent systemic and ophthalmic inflammation she was changed to adalimumab; however, this was also unsuccessful and methotrexate was added. By the age of 18 she had been steroid-dependent for 2 years and could not reduce daily prednisolone below 15mg without a deterioration in her left eye posterior scleritis with visual acuity compromise including episodes of complete visual loss causing high levels of anxiety. Due to the pain and deterioration in vision she struggled with her studies and school attendance, and withdrew from her passion for competitive sailing. With ongoing sight-threatening inflammatory changes she was referred for further tertiary assessment in 2019.

During the following 4 months treatment was escalated rapidly. Methotrexate dose was increased, and adalimumab frequency reduced to weekly. There was a limited response, with further episodes of sight deterioration as a result of flares in inflammation. Response to tocilizumab treatment was seen after only two doses with good control of scleritis by 3 months of treatment when steroids were successfully weaned and stopped. Over 18 months of tocilizumab therapy the patient has only required one course of topical steroids for mild ocular inflammation which resolved without any other treatment required. She has successfully completed her degree, can complete daily gym training sessions and participate in regular sailing competitions.

**Case report - Discussion:**

Posterior scleritis is the most common scleritis in children and can be associated with anterior uveitis, concurrent anterior scleritis, disc swelling or retinal striae. Posterior scleritis has a higher rate of complications therefore is treated aggressively. Refractory cases such as this require biologic therapy and rituximab is often used. Despite the preference of two adult eye units for treatment with rituximab the rationale for tocilizumab included; recent high quality studies showing successful treatment of inflammatory bowel disease, its known benefit for anterior uveitis and case studies in adults with posterior scleritis.

Tocilizumab is a recombinant monoclonal antibody that causes a blockade of interleukin-6 receptors. It is currently only approved by the Food and Drug Administration (FDA) for use in children with polyarticular or systemic onset Juvenile Idiopathic Arthritis (JIA). Our patient had a very positive experience with this drug, no side effects and rapid clinical improvement seen. As a result her quality of life and mental health improved quickly.

**Case report - Key learning points:**

This is a case of refractory, sight-threatening, steroid-dependent posterior scleritis on a background of inflammatory bowel disease and arthritis. As a result of this case our team reviewed current literature from other paediatric populations and adults with scleritis, informing the clinical decision to proceed with tocilizumab after control was unsuccessful with previous agents. The remarkable response demonstrated for our patient highlights the value tocilizumab can offer to the treatment options for similar refractory cases. This adds to the growing positive data published surrounding tocilizumab in children but further studies in paediatric populations are required to evaluate this in greater detail.

